# Postpartum ovarian vein thrombosis manifesting as acute appendicitis: a case report

**DOI:** 10.1186/s13256-021-03102-y

**Published:** 2021-10-25

**Authors:** Christos Tsitlakidis, Khalil Ibrahim Salim Al Ajmi, Alya Yousuf Al Madhani, Adel Hassan Ahmidat

**Affiliations:** 1 York Teaching Hospital, NHS Foundation Trust, Scarborough, UK; 2 Surgical Department, Sultan Qaboos University Hospital, Muscat, Oman; 3 Royal Hospital, Muscat, Oman

**Keywords:** Ovarian vein, Thrombosis, Postpartum, Acute appendicitis, Appendicectomy

## Abstract

**Background:**

Postpartum ovarian thrombosis is an uncommon condition. It appears with the nonspecific, predominantly right-sided abdominal symptoms and must be differentiated from other acute visceral conditions. If left untreated, postpartum ovarian thrombosis can have severe consequences, including sepsis, pulmonary embolism, and even death. Momentarily, there are no specific guidelines for postpartum ovarian thrombosis management. We present a case of postpartum ovarian thrombosis admitted to our hospital with symptoms of acute appendicitis.

**Case presentation:**

A 39-year-old Omani obese multiparous woman of Afro-Arab origin was admitted with acute symptoms, mainly abdominal pain, fever, and vomiting 1 week postpartum. Clinical picture and biochemical profile did not exhibit a recognizable pattern. Ultrasonography excluded retained products of conception. Computerized scan for abdomen and pelvis with oral and intravenous contrast reported a dilated tubular structure in the right adnexa extending up to the right renal hilum level with surrounding inflammation. Those findings were consistent with the thrombophlebitis of the right ovarian vein. Blood cultures and sensitivity showed group A β-hemolytic streptococci sensitive to penicillin G and clindamycin. The patient was treated successfully with antibiotics and therapeutic anticoagulants and discharged home 3 days later; follow-up was arranged.

**Conclusion:**

This pathology is an exceptional entity in Oman. Therefore, awareness of this unique condition is required so that clinicians will be vigilant, exploring similar cases with imaging and avoiding unnecessary surgical interventions.

## Background

Ovarian vein thrombosis is a complication during pregnancy and the postpartum period. Postpartum (puerperal) ovarian vein thrombosis (POVT) appears in 0.05–0.18% after vaginal delivery [1] and 1–2% after caesarean section [2–4]. The underlying physiology driving the thrombus formation can be seen within Virchow’s triad: venous stasis, vessel wall damage, and the presence of a hypercoagulable state [5]. Apart from pregnancy and the puerperium, where a hypercoagulability state exists due to the surge of estrogens [4], Virchow’s conditions also arise from major pelvic surgery, inflammatory disease, coagulopathy, and malignancy, thus addressing the disease over a broad age population [6]. Furthermore, nine idiopathic OVT cases have been acknowledged [7]. Anatomically, there is a notable predilection for the right ovarian vein to be thrombosed. Valve incompetence and absence of retrograde flow both promote thrombus extension in the right ovarian vein. During puerperium, diminished fibrinolytic activity and elevation of coagulation factors aid in hemostasis and prevent blood loss [8]; besides, the uterus undergoes physiological dextrorotation and predisposes the right vein to thrombosis due to the acute angle formed where it enters the inferior vena cava [9, 10]. As a result, in 80–90% of postpartum cases, there is a unilateral right-sided involvement, in contrast with other etiologies where OVT entanglement was observed on either side or bilaterally [11]. Clinically, OVT asymptomatic appearance in most cases with malignancy is in contrast with the typical presentation during the postpartum period: fever with chills, diffuse pelvic and right iliac fossa pain, and a tender, rope-like mass palpable in the lower abdomen [6], and leukocytosis 2–15 days postpartum. Fatal cases have been reported [6] from sepsis, thrombosis of the inferior vena cava, renal veins, and pulmonary embolism.

## Case report

A 39-year-old woman, Omani of Afro-Arab origin, para 5, obese, was admitted 7 days after spontaneous vaginal delivery with right-sided lower abdominal pain, nausea and vomiting (food content, no blood), and fever (38 °C) with chills. The onset of the symptoms was 2 days of feeling unwell and having generalized abdominal pain. She lost her appetite and was unable to eat. Other symptoms included four episodes of loose motions with watery stools on admission, lethargy, and difficulty standing. In addition, she reported a mild dry cough for 4 days and a sore throat. She had an uneventful vaginal delivery of a live term male; her pregnancy had been complicated by chronic hypertension, well controlled on labetalol and gestational diabetes managed with metformin. She had no history of thrombophilia. Vitals on admission showed a respiratory rate of 20 breaths/minute, SaO_2_ 99% on room air, temperature 37.1 °C, pulse rate of 130 beats/minute, and 129/69 mmHg blood pressure.

Blood studies on admission showed white blood cells (WBC) and neutrophil count normal, lactate 2.5 mmol, prolonged activated partial thromboplastin time (APTT) of 40.5 (normal value 26.4–38.9) seconds, derived fibrinogen 5.18 (normal value 1.6–4) g/l, and thrombin time of 17.5 (normal value 14.3–17.8) seconds. International normalized ratio (INR) was 1.25 (normal value 0.82–1.05). The liver function profile was normal. The renal function panel revealed a picture of acute kidney injury. D-dimer was not done.

On abdominopelvic examination, there was abdominal tenderness over the right iliac fossa, with rebound tenderness. Rovsing’s sign was positive. In addition, there was a uterine subinvolution (20 weeks size). No calf tenderness or swelling was noted on leg examination to suggest deep venous thrombosis. Similarly, speculum examination was unremarkable; lochia was present in small amounts, without foul odor. Surgical consultation ruled out the possibility of acute appendicitis.

Abdominopelvic ultrasound reported an intramural fibroid and congested veins surrounding the uterus in keeping with postpartum status. The appendix and the ovaries have not been visualized, and there were no retained products of conception. In addition, the patient experienced severe probe tenderness during the transvaginal scan (TVS).

**Fig. 1:**
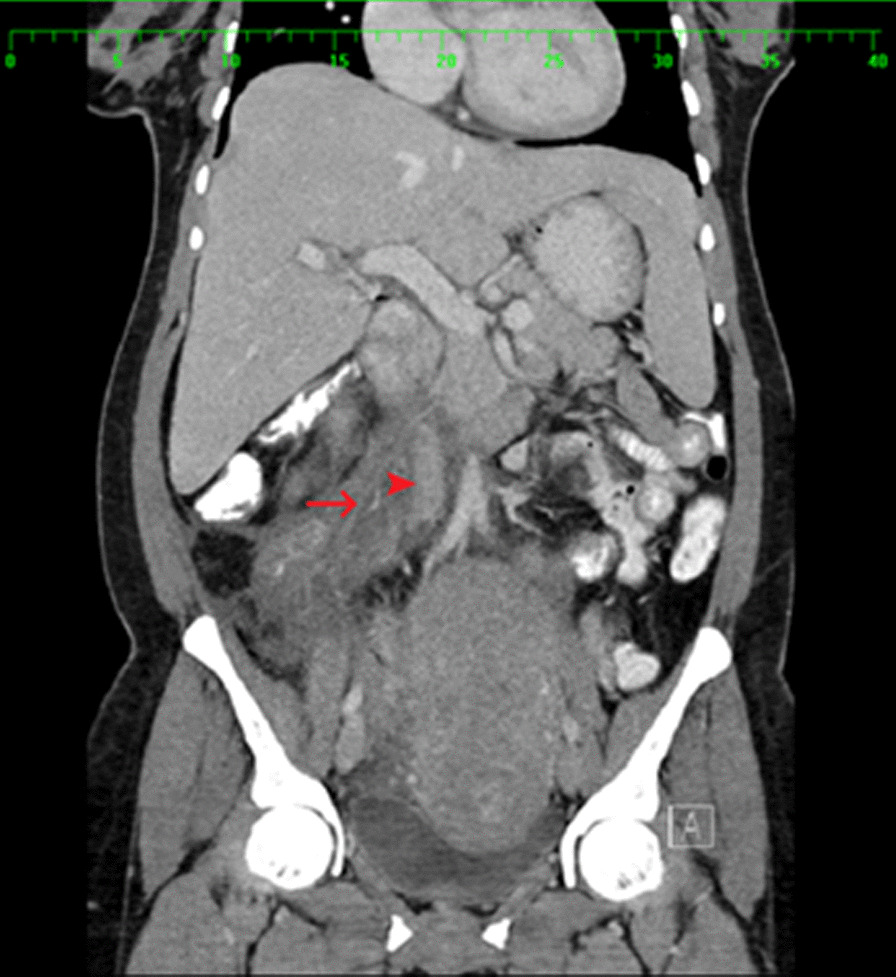
Arrow: thrombosed right ovarian vein. Arrowhead: inferior vena cava. Figs. 1, 2, 3, 4: Halima Al-Amri (2020). Radiologic images of the patient, Sultan Qaboos University Hospital, Muscat, Oman

**Fig. 2.**
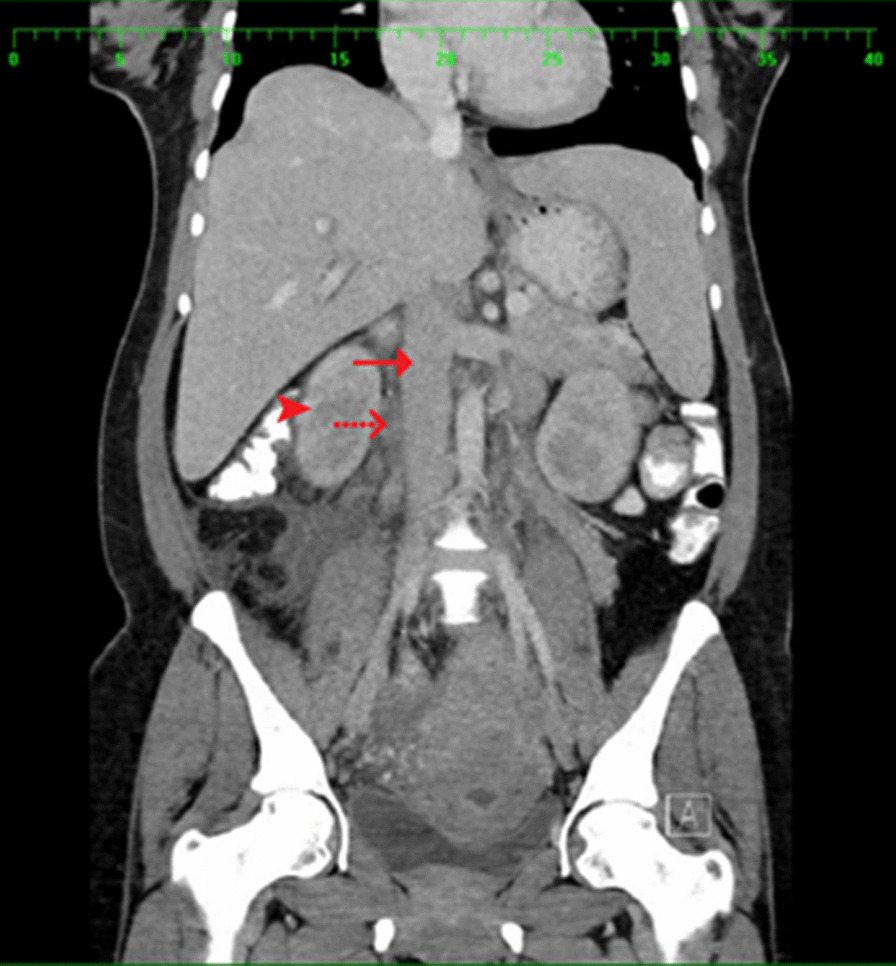
Arrow: inferior vena cava. Dashed arrow: thrombosed right ovarian vein. Arrowhead: right kidney. Figs. 1, 2, 3, 4: Halima Al-Amri (2020). Radiologic images of the patient, Sultan Qaboos University Hospital, Muscat, Oman

**Fig. 3:**
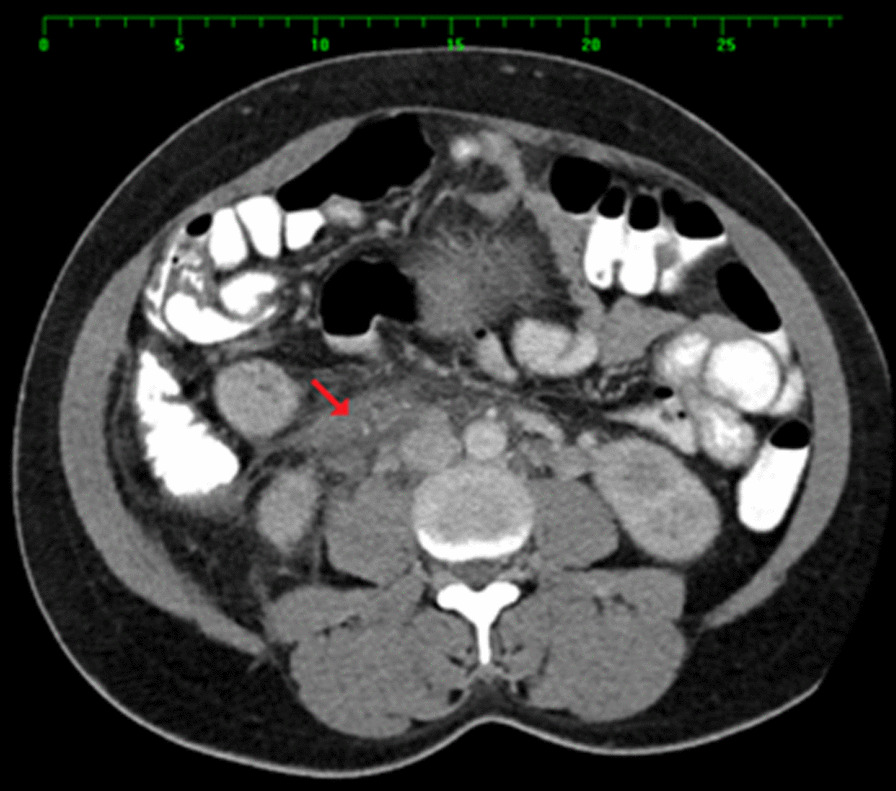
Arrow points to right ovarian vein. Figs. 1, 2, 3, 4: Halima Al-Amri (2020). Radiologic images of the patient, Sultan Qaboos University Hospital, Muscat, Oman

**Fig. 4:**
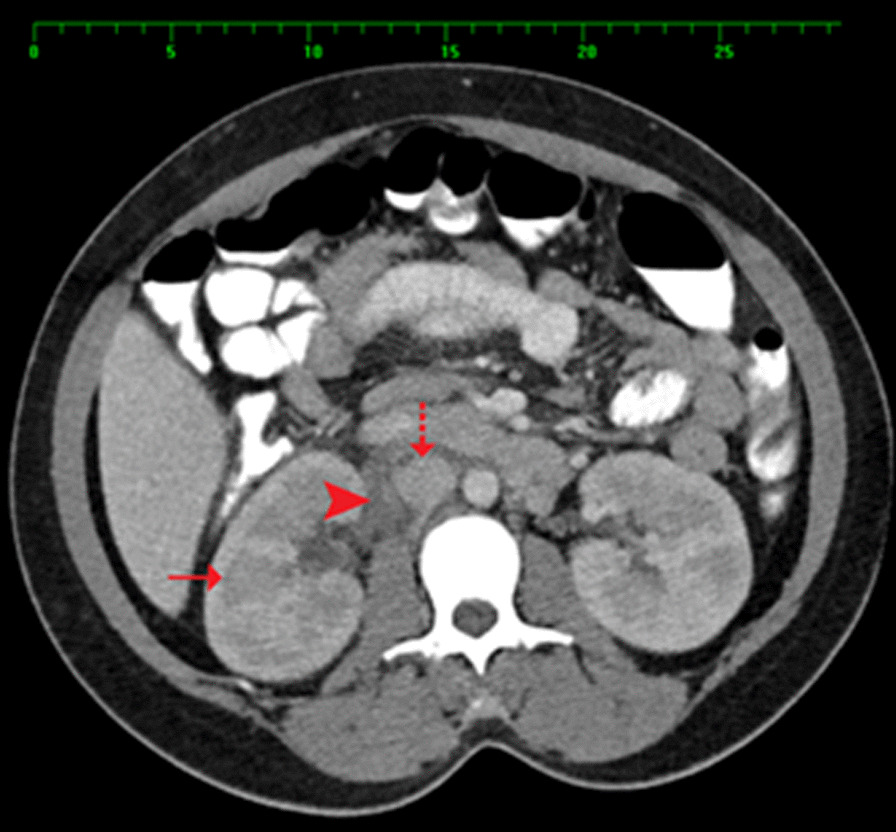
Arrow points to the right kidney. Arrowhead: thrombosed right ovarian vein. Dashed arrow: inferior vena cava. Figs. 1, 2, 3, 4: Halima Al-Amri (2020). Radiologic images of the patient, Sultan Qaboos University Hospital, Muscat, Oman

An abdominal CT scan with oral and intravenous contrast revealed a tubular-like structure (Figs 1, 2, 3, 4) extending from the pelvis to the abdomen up to the right renal hilum level, not separable from the right adnexa, measuring 11 × 3 cm in size. This mass had heterogeneous density with surrounding significant fat stranding, extending from the retroperitoneal area to the renal level. This dilated tubular structure with the surrounding inflammation was typical for thrombophlebitis of the right ovarian vein. The appendix was seen in the right iliac fossa with a normal appearance; it measured 7 mm in cross-section image, and there was intraluminal gas seen with contrast within the lumen. Surgical clips were visible at the site of previous laparoscopic cholecystectomy. Echocardiography ruled out infective endocarditis and showed normal cardiac function.

Therapeutically, this patient was administered vancomycin and tazocin, which then changed to penicillin G and clindamycin following blood culture and sensitivity results unveiling the growth of group A β-hemolytic streptococci.

Hematology team opinion was obtained, and the patient started therapeutic anticoagulation with enoxaparin sodium and warfarin overlap (target INR 2–3). She was planned to continue for a minimum of 3 months, and the investigation of other causes for thrombosis was deferred as it was likely provoked by postpartum.

The patient clinically improved and was eventually discharged home 3 days later in good condition. Her discharge medication was warfarin 3.5 mg once daily for a total of 3 months and a full course of antibiotics for 1 week; follow-up was arranged, 6 weeks following discharge from the hospital with a view of repeat imaging if deemed necessary. The patient continued to follow up in a secondary care hospital rather than this tertiary center.

## Discussion

Ovarian vein thrombosis is rare; compared with deep vein thrombosis occurrence of 1 in 1000 adults (DVT), incidence rates are 60-fold lower [12]. Symptomatic POVT appears in 0.01–0.03% after vaginal delivery [13]. Only two POVT cases have been documented in Oman; these were treated initially as a dilated ureter [14]. Initial presentation with pulmonary embolism was in 6% of patients with OVT compared with 16% of those with DVT [12]. Predisposing factors include cancer, hormonal stimulation, and hospitalization; preceding surgery and personal history of thromboembolism are independent factors for recurrence but with no difference in the overall survival for those treated with anticoagulants [12]. In cases with malignancy, the OVT association has detrimental effects on survival rates from the disease [12]. Aggravating symptoms in OVT can occur from delay in diagnosis; extension of the thrombus into the inferior vena cava (25–30%) or left renal vein [13]; sepsis and pulmonary embolism appear in one out of four cases, raising the mortality rate to 4% [4]. Differential diagnoses, apart from endometritis, a commonly mistaken diagnosis [15], include a hydroureter or appendix, and this is attributed to the gas-filled bowel located in the same region [14, 16]; ovarian torsion and ovarian cyst are also considered [4]. OVT poses a diagnostic puzzle that does not fit into a recognizable pattern and requires radiological imaging [6]. D-dimer (DD) has poor diagnostic value during pregnancy and postpartum; however, a cut-off at 500 ng ml (−1) DD measurement has been reported to be useful 4 weeks after delivery [17]. The choice of initial imaging technique largely depends on test availability and the clinical picture [13]. Ultrasound may be used as first-line imaging, and if not obscured by bowel gas, it will show the thrombosed vein, enlarged with an intraluminal echogenic mass [2]. The supplementary use of Doppler with ultrasound yields a higher sensitivity in detecting the condition [13]. On color Doppler, there will be reduced or no flow within the lumen of the vein and probably increased flow in the perivascular region due to inflammation [2]. Additional imaging is required to investigate for ovarian vein thrombosis as ultrasound does not exclude it [18]. Intravenous contrast-enhanced CT allows accurate delineation of OVT [19]. Complications of POVT include sepsis, ureteral compression, pulmonary embolism, and propagation of the thrombus to the inferior vena cava and renal veins [1]. MRI is a tool of choice and has the highest sensitivity and specificity [13]; it provides the benefit of avoiding radiation and assessing the superior extent of the thrombus into the inferior vena cava or the renal veins [2].

Medical treatment includes the use of anticoagulation alone, which presented similar results with combined therapy [20], in contrast with previous beliefs where the addition of heparin to antimicrobial therapy did not result in better outcomes [21]. The most favorable treatment of choice is a combination of anticoagulation and antibiotics [18]. Antibiotics choices are like those used in septic pelvic thrombophlebitis and consist of intravenous agents like imipenem and cilastatin, ampicillin and sulbactam, clindamycin, and gentamicin single-drug therapy with a second- or third-generation cephalosporin [22]. Non-evidence-based drug prescription should be avoided; such practice has led to antibiotic resistance, a worldwide peril especially for immunosuppressed patients [23]. The use of anticoagulation with heparin precludes the passages and propagation of septic emboli and quickens fever’s abatement [22]. The duration of antibiotic treatment is 48–72 hours. Anticoagulation is recommended for 7–10 days or at least 7–10 days after fever resolution.

Low-molecular-weight heparin (LMWH) and unfractionated heparin are comparable in efficacy. LMWH is preferable over unfractionated heparin as it has a favorable adverse event profile compared with unfractionated heparin [18]. Specific therapeutic interventions are indicated in patients with recurrent pulmonary embolism, poor compliance, and contraindicated anticoagulation. Interventional procedures include thrombectomy and filter placement in the inferior vena cava. Surgical treatment includes vein ligation, oophorectomy, and hysterectomy [13, 15, 18, 24]. Commencement of treatment with unfractionated heparin followed by warfarin or LMWH has been traditionally used as an anticoagulation regimen treatment that ranges between 3 and 6 months [26]. Three months of anticoagulation therapy were seen with no thrombosis recurrence over a median follow-up of 40 months [20]. Although there is no agreed consensus for the length of anticoagulation therapy and POVT has a high rate of resolution after short treatment [20], 3-month treatment with anticoagulants is suggested in cases of symptomatic postpartum OVT, with the addition of antibiotics [13]. A small thrombotic pelvic vein can be treated with a short therapy lasting 2–3 weeks [25]. Besides, asymptomatic POVT can be managed without anticoagulants unless there is evidence from imaging of thrombus extension or pulmonary embolism occurrence [13]. Warfarin is the better alternative as it is more convenient with no injections and safe breastfeeding [16]. It was reported that the recurrence risk of postpartum ovarian vein thrombosis in future pregnancies is low; however, there is a paucity of data in this regard [18]; nonetheless, patients with a history of thrombotic events necessitate anticoagulant cover in future pregnancies, and thrombophilia screening should be evaluated in non-pregnancy related cases.

## Conclusion

The condition presents a differential diagnosis in cases of inexplicable postpartum abdominal pain with a worsening clinical condition. Singular treatment with antibiotics could lead to severe morbidity from septic embolism of renal veins and occasional death from a pulmonary embolus. A multidisciplinary approach to suspected ovarian vein thrombosis is paramount for optimal postnatal outcomes and involves obstetricians, hematologists, microbiologists, and radiology specialists. Awareness and clinical knowledge are crucial in treating accordingly and avoiding unnecessary surgical interventions.

## Data Availability

Supporting data are available on request

## Abbreviations

APTT: Activated partial thromboplastin time
CT: Computerized tomography scan
INR: International normalized ratio
LMWH: Low-molecular-weight heparin
MDCT: Multiple detector computerized tomography scan
MRI: Magnetic resonance scan
OVT: Ovarian vein thrombosis
POVT: Postpartum (puerperal) ovarian vein thrombosis
TVS: Transvaginal scan
WBC: White blood cells
